# Nonsurgical pancreatic cancer: the role of radiotherapy in prolonging survival – a retrospective cohort study in the SEER database

**DOI:** 10.1097/JS9.0000000000001885

**Published:** 2024-06-26

**Authors:** Yupeng Di, Jiazhao Song, Zhijia Sun, Yingjie Wang, Lingling Meng

**Affiliations:** a Department of Radiation Oncology, Air Force Medical Center, PLA, Beijing, People's Republic of China; b Department of Radiation Oncology, Senior Department of Oncology, The Fifth Medical Center of PLA General Hospital, Beijing, People’s Republic of China

**Keywords:** locally advanced pancreatic cancer, radiotherapy, SEER database, survival benefits

## Abstract

**Background:**

Limited research has compared external beam radiotherapy (RT) to non-RT in patients with nonsurgical locally advanced pancreatic cancer (LAPC). Therefore, this study investigates the impact of RT on overall survival (OS) in patients with nonsurgical LAPC in a real-world context.

**Methods:**

The authors conducted an analysis of patients with nonsurgical LAPC using data from the Surveillance, Epidemiology, and End Results (SEER) database. This analysis involved the utilization of Kaplan–Meier survival curves and multivariable Cox regression analyses.

**Results:**

A total of 5413 individuals with nonsurgical LAPC were included in this analysis. Among them, 2320 (42.9%) received RT, while 3093 (57.1%) underwent non-RT treatment. The median OS was 12.0 months for the RT group and 9.0 months for the non-RT group, with a statistically significant difference (*P*<0.001). Multivariate analysis revealed that RT had a statistically significant impact on OS (HR, 0.86; 95% CI: 0.81–0.91; *P*<0.001). Propensity score matching analysis confirmed a statistically significant association of RT with improved OS (HR, 0.84, 95% CI: 0.79–0.90; *P*<0.001). These results remained consistent after conducting sensitivity analyses, subgroup analyses, and propensity score matching.

**Conclusion:**

The study findings suggest that RT could be advantageous for patients with nonsurgical LAPC. Further investigations are warranted to explore the relationship between RT and OS.

## Introduction

Highlights

- Palliative radiotherapy for patients with nonsurgical locally advanced pancreatic cancer is not supported by prospective randomized controlled clinical trials.
- Our results suggest that palliative radiotherapy is feasible for patients with nonsurgical locally advanced pancreatic cancer.
- These results remained consistent after performing sensitivity analyses, subgroup analyses and propensity score matching.
- Our study’s findings will inform prospective, highly informative, and impactful clinical studies.

Nonsurgical locally advanced pancreatic cancer (LAPC) typically presents a bleak prognosis; however, recent data indicate an improvement in its 3-year survival rate^1^. At present, radical surgery remains the sole curative option for pancreatic cancer. However, the insidious onset of this ailment results in over 80% of patients being diagnosed at advanced stages, rendering surgical intervention unattainable. Furthermore, the risk of recurrence remains unacceptably high even postresection, consequently constraining the survival rate within this patient cohort^2^. Therefore, to extend patient survival and improve quality of life, it is necessary to explore and apply more effective treatment methods.

Systemic chemotherapy is the standard treatment for LAPC^3^. Previous studies have shown that systemic therapy plays an important role in LAPC^4^. Preoperative chemotherapy and radiotherapy (RT) can improve surgical resection rates, and conversion surgery is increasingly being studied^5^. The results of a meta-analysis showed that in LAPC^6^, first-line treatment with the FOLFIRINOX regimen led to a successful conversion to surgery in 25.9% of patients, meaning that about 3/4 of patients could not undergo surgery. The majority of patients who did not undergo surgery received chemotherapy. A retrospective cohort study that confirmed the survival benefit of RT combined with chemotherapy over chemotherapy alone using PSM analysis was inadequate in that it did not analyze the survival of patients treated with RT alone^7^. With this in mind, our study focused on the relationship between RT and nonsurgical LAPC.

Though multiple investigations have revealed the local control and survival advantages associated with RT in nonsurgical LAPC patients^8,9^, the dearth of randomized trials and retrospective reviews examining the utility of RT in this context is noteworthy^10,11^.

In light of this gap, we undertook a comprehensive study employing data from the Surveillance, Epidemiology, and End Results (SEER) database. The primary objectives of our study were to determine whether patients afflicted with nonsurgical LAPC who received RT experienced improved 3-year overall survival (OS) compared to those who did not receive RT and to assess the potential effect of RT on OS in nonsurgical LAPC patients.

## Materials and methods

### Study population

We conducted a retrospective analysis involving patients diagnosed with nonsurgical LAPC who had received either RT or non-RT treatment. Data were taken from the SEER version 8.4.2 database, which includes information from the 18 registries in the SEER database from 1975 to 2022. During the period from 2004 to 2015, the database represented ~28% of the United States population. The SEER program follows standardized data collection procedures and exclusively provides de-identified data^12–14^. One of the authors obtained permission to access the database (username: 15395-Nov2022). Our study adhered to the Strengthening the Reporting of cohort, cross-sectional, and case–control studies in Surgery (STROCSS) criteria^15^.

As all the data used in this study were derived from publicly available sources within the SEER database, there was no requirement for informed consent or ethical approval.

Patients diagnosed with LAPC were identified and categorized based on a combination of anatomical and histological codes from the International Classification of Diseases of Oncology, 3rd edition (ICD-O-3). The ICD-O-3 site codes for LAPC (T4NXM0) encompassed C25.0–C25.4 and C25.7–C25.9, while the histological codes included 8000, 8001, 8010, 8012, 8013, 8020-8022, 8030-8033, 8041, 8046, 8070, 8071, 8140, 8141, 8160, 8162, 8201, 8246, 8255, 8260, 8310, 8430, 8440, 8441, 8452,8453, 8470, 8471, 8480, 8481, 8490, and 8500.

We specifically selected patients diagnosed between 2004 and 2015 ensuring their histological confirmation and that they had no prior surgical procedures. These individuals were then categorized based on whether they had received RT. To ensure data integrity, we excluded patients lacking a histological diagnosis or autopsy confirmation of LAPC, those with a survival duration of less than 2 months following diagnosis, and patients with primary sites that underwent resected tumor surgery. We also excluded patients who were recommended RT but for whom it remained uncertain whether this treatment was administered.

### Statistical analyses

We conducted multivariate Cox regression analyses to explore the independent associations between RT and enhanced prognosis. The primary trial endpoint was defined as the 3-year OS. Survival curves were constructed using Kaplan–Meier analysis, and the log-rank test compared RT and non-RT patient groups. Subgroup analyses were conducted to investigate the relationship between radiation status and survival outcomes, adjusting for potential confounding factors. Interaction tests within Cox proportional hazards models compared hazard ratios (HRs) between the examined subgroups.

We performed a descriptive analysis on all study participants. Categorical variables are presented as proportions and percentages (%), while continuous data are reported as medians with interquartile ranges. Comparisons between variables were conducted using *χ* ^2^ tests for categorical variables and one-way analysis of variance for normally distributed continuous variables. For skewed distributions, the Kruskal–Wallis test was utilized.

All statistical analyses were executed using R version 4.3.1 (The R Foundation) and comprehensive statistical analysis of the collected data was conducted using SPSS 25.0 (IBM Corporation). Statistical significance was established at a two-tailed *P*-value of less than 0.05.

### Sensitivity analyses

Retrospective studies inherently introduce selection bias due to baseline characteristic imbalances among participants. This bias may have affected the survival outcomes of patients who received RT and those who did not in the current study. Propensity score matching (PSM) was employed to effectively mitigate selection bias by aligning the treated and nontreated groups based on measurable covariates.

In this investigation, the patients’ receipt of RT or non-RT treatment was considered the dependent variable. Multivariate models included variables deemed potential confounders based on prior studies and clinical expertise. Chemotherapy, age, sex, year of diagnosis, marital status, race, tumor grade, and tumor stage were the factors considered for PSM. A 1:1 closest neighbor matching technique was employed, with a caliper width of 0.1. The aforementioned statistical approach facilitated comparisons between participants who did and did not receive RT before and after PSM. The degree of PSM was assessed using standardized mean differences, with a criterion of <0.1 signifying an acceptable level of significance.

## Results

### Population

**Figure 1:**
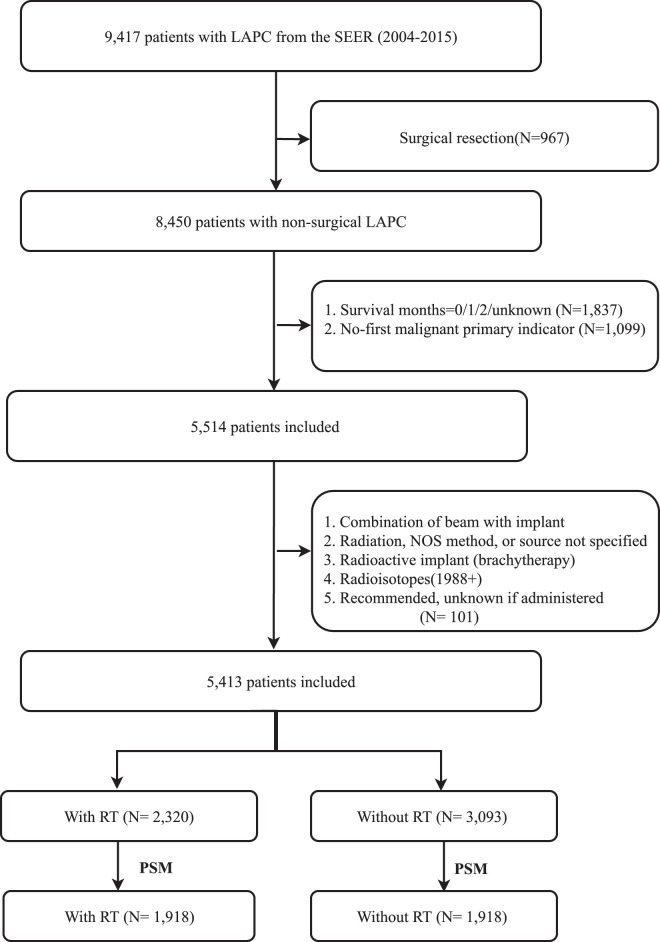
Flowchart for study enrollment. RT, radiotherapy; PSM, propensity score matching; SEER, Surveillance, Epidemiology, and End Results; LAPC, locally advanced pancreatic cancer

Between 2004 and 2015, the SEER database identified 9417 patients with LAPC according to the specified criteria. After excluding individuals with missing or inappropriate data, including diagnoses, surgical resection, initial malignant primary indicator, and survival duration (as evaluating efficacy for survival times less than 3 months is impractical), the study cohort was reduced to 5514 eligible individuals. Patients who had undergone unknown radiation treatment or had received a radioisotope or implant were also excluded. Consequently, the final study cohort consisted of 5413 patients who underwent analysis. The study enrollment process is illustrated in Figure 1.

### Baseline characteristics

Table 1 presents a summary of the baseline demographic information for all participants. Among the total cohort, 2320 individuals (42.9%) underwent RT, while 3093 (57.1%) were classified as non-RT recipients. The median age of the study population was 65.9 years, with a range spanning from 26 to 100 years. The average survival duration was 13.2 months. Notably, significant statistical differences were observed in age (*P*<0.001), year of diagnosis (*P*<0.001), marital status (*P*<0.001), and chemotherapy status (*P*<0.001) based on the RT treatment status. Additionally, treatment type correlated with N stage (N0, N+, N unknown) and tumor grade (I/II, III/IV, unknown).

**Table 1:** Baseline characteristics of patients who nonsurgical pancreatic ductal adenocarcinoma treated with nonradiotherapy and radiotherapy before and after propensity score matching.

Variables	Patient characteristics (raw data)				After PSM			
	Total (*n*=5413)	Non-RT (*n*=3093)	RT (*n*=2320)	*P*	Total (*n*=3846)	Non-RT (*n*=1923)	RT (*n*=1923)	*P*
Age, *n* (%)				< 0.001				1.000
＜65	2474 (45.7)	1268 (41.0)	1206 (52.0)		1855 (48.2)	928 (48.3)	927 (48.2)	
≥65	2939 (54.3)	1825 (59.0)	1114 (48.0)		1991 (51.8)	995 (51.7)	996 (51.8)	
Sex, *n* (%)				0.754				0.897
Male	2720 (50.2)	1548 (50.0)	1172 (50.5)		1955 (50.8)	975 (50.7)	980 (51.0)	
Female	2693 (49.8)	1545 (50.0)	1148 (49.5)		1891 (49.2)	948 (49.3)	943 (49.0)	
Year of diagnosis, *n* (%)				< 0.001	0.365			
2004–2007	1527 (28.2)	789 (25.5)	738 (31.8)		988 (25.7)	476 (24.8)	512 (26.6)	
2008–2011	1865 (34.5)	1009 (32.6)	856 (36.9)		1374 (35.7)	689 (35.8)	685 (35.6)	
2012–2015	2021 (37.3)	1295 (41.9)	726 (31.3)		1484 (38.6)	758 (39.4)	726 (37.8)	
Race, *n* (%)				0.995				0.758
White	4203 (77.6)	2401 (77.6)	1802 (77.7)		2975 (77.4)	1492 (77.6)	1483 (77.1)	
Other	1210 (22.4)	692 (22.4)	518 (22.3)		871 (22.6)	431 (22.4)	440 (22.9)	
Marital, *n* (%)				0.003				0.554
Married	3120 (57.6)	1729 (55.9)	1391 (60.0)		2299 (59.8)	1140 (59.3)	1159 (60.3)	
Other	2293 (42.4)	1364 (44.1)	929 (40.0)		1547 (40.2)	783 (40.7)	764 (39.7)	
Grade, *n* (%)				0.195				0.640
I/II	878 (16.2)	486 (15.7)	392 (16.9)		611 (15.9)	296 (15.4)	315 (16.4)	
III/IV	626 (11.6)	344 (11.1)	282 (12.2)		468 (12.2)	231 (12.0)	237 (12.3)	
Unknown	3909 (72.2)	2263 (73.2)	1646 (70.9)		2767 (71.9)	1396 (72.6)	1371 (71.3)	
Tumor Site, *n* (%)				0.027				0.874
Head	2960 (54.7)	1660 (53.7)	1300 (56.0)		2106 (54.8)	1061 (55.2)	1045 (54.3)	
Body/Tail	1243 (23.0)	701 (22.7)	542 (23.4)		903 (23.5)	447 (23.2)	456 (23.7)	
Unknown/Over	1210 (22.4)	732 (23.7)	478 (20.6)		837 (21.8)	415 (21.6)	422 (21.9)	
N stage, *n* (%)				0.012				0.879
0	3292 (60.8)	1855 (60.0)	1437 (61.9)		2318 (60.3)	1165 (60.6)	1153 (60.0)	
1	1796 (33.2)	1027 (33.2)	769 (33.1)		1314 (34.2)	654 (34.0)	660 (34.3)	
Unknown	325 ( 6.0)	211 ( 6.8)	114 (4.9)		214 ( 5.6)	104 (5.4)	110 (5.7)	
Chemotherapy, *n* (%)				< 0.001				1.000
No	1237 (22.9)	1100 (35.6)	137 (5.9)		274 ( 7.1)	137 (7.1)	137 (7.1)	
Yes	4176 (77.1)	1993 (64.4)	2183 (94.1)		3572 (92.9)	1786 (92.9)	1786 (92.9)	

All normally distributed and skewed continuous variables are presented as medians with interquartile ranges. Categorical variables are expressed as frequencies and percentages (%).

*P* values: For baseline characteristic analysis, the statistical differences among the quartiles of radiation were tested using a one-way analysis of variance for continuous variables and *χ* ^2^ tests for categorical variables. Statistical significance was set at *P*<0.05.

LAPC, locally advanced pancreatic cancer; PSM, propensity score matching; Ref, referent; RT, radiotherapy.

### Survival analysis

**Figure 2:**
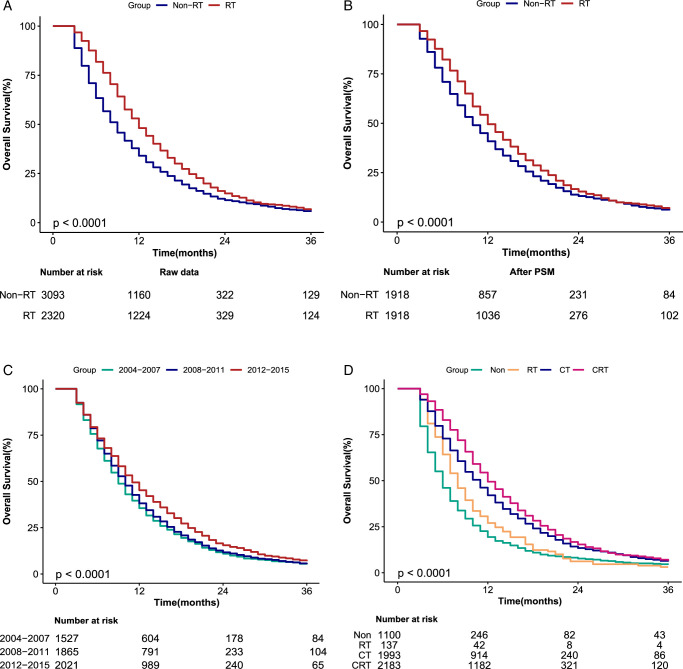
Overall survival curves of cases with nonsurgical LAPC according to RT (A), RT after PSM (B), the year of diagnosis (C) and treatment (D). A Log-rank test was utilized to compare curves, and significance presented on each panel. CT, chemotherapy; CRT, chemotherapy combined with radiotherapy; LAPC, locally advanced pancreatic cancer; PSM, propensity score matching; RT, radiotherapy.

The survival duration for the entire cohort was assessed with a 36-month observation endpoint, meaning participants surviving beyond 36 months were categorized as having a 36-month survival duration. The median OS was 12.0 months for patients who received RT and 9.0 months for those without RT treatment (*P*<0.0001). Examination of the treatment modalities revealed statistically significant differences in survival within the first 3 years of treatment initiation (*P*<0.001). The 1-year survival rates were 48.0% for the RT group and 34.0% for the non-RT group. At 2 years, survival rates were 14.8% for the RT group and 11.4% for the non-RT group. The 3-year survival rates were 6.3% for the RT group and 5.6% for the non-RT group. Most patient deaths occurred within 24 months of treatment (Fig. 2A).

#### Univariate OS analyses

As indicated in Table 2, patients who received RT (HR, 0.77; 95% CI: 0.72–0.81; *P*<0.001) or chemotherapy (HR, 0.57; 95% CI: 0.53–0.61; *P*<0.001) experienced a significant reduction in mortality. Age (≥65 years: HR, 1.21; 95% CI: 1.15–1.28) was inversely related to OS. In this study, individuals without a marital status defined as ‘other’ (HR, 1.13; 95% CI: 1.07–1.19) were associated with OS. No statistically significant differences in mortality were observed based on sex (female HR, 1.00; 95% CI: 0.95–1.06) or race (other-race HR, 0.99; 95% CI: 0.93–1.06). Patients diagnosed with cancer between 2008 and 2011 (HR, 0.96; 95% CI: 0.90–1.03) did not exhibit a substantially improved OS compared to those treated between 2004 and 2007. In contrast, patients diagnosed between 2012 and 2015 displayed the most substantial reduction in mortality (HR, 0.84; 95% CI: 0.78–0.90). When compared to grade I/II, patients with grade III/IV (HR, 1.45; 95% CI: 1.30–1.61) and unknown grade (HR, 1.13; 95% CI: 1.04–1.22) disease exhibited a higher risk of mortality. Patients with stage N+ (HR, 1.10; 95% CI: 1.03–1.17) or N unknown (HR, 1.14; 95% CI: 1.02–1.29) disease had worse outcomes than those with stage N0.

**Table 2:** Prognostic factors for overall survival using univariate and multivariate analysis before and after propensity score matching.

	Before PSM				After PSM			
	Univariate analysis		Multivariate analysis		Univariate analysis		Multivariate analysis	
Variable	HR (95% CI)	*P*	HR (95% CI)	*P*	HR (95% CI)	*P*	HR (95% CI)	*P*
RT								
No	Ref		Ref		Ref		Ref	
Yes	0.77 (0.72–0.81)	<0.001	0.86 (0.81–0.91)	<0.001	0.85 (0.79–0.90)	<0.001	0.84 (0.79–0.90)	<0.001
Age								
＜65			1.19 (1.12–1.25)					
≥65	1.21 (1.15–1.28)	<0.001		<0.001	1.14 (1.07–1.22)	<0.001	1.16 (1.08–1.24)	<0.001
Sex								
Male	Ref		Ref		Ref		Ref	
Female	1.00 (0.95–1.06)	0.929	0.96 (0.91–1.02)	0.181	0.96 (0.90–1.02)	0.198	0.93 (0.86–0.99)	0.027
Year of diagnosis								
2004–2007	Ref		Ref		Ref		Ref	
2008–2011	0.96 (0.90–1.03)	0.236	0.98 (0.91–1.05)	0.493	0.98 (0.90–1.07)	0.641	0.99 (0.91–1.07)	0.758
2012–2015	0.82 (0.77–0.88)	<0.001	0.84 (0.78–0.90)	<0.001	0.78 (0.72–0.85)	<0.001	0.80 (0.73–0.87)	<0.001
Race								
White	Ref		Ref		Ref		Ref	
Other	0.99 (0.93–1.06)	0.800	1.01 (0.94–1.08)	0.835	1.01 (0.93–1.09)	0.857	1.01 (0.93–1.10)	0.769
Marital								
Married	Ref		Ref		Ref		Ref	
Other	1.13 (1.07–1.19)	<0.001	1.10 (1.04–1.16)	0.001	1.1 (1.03–1.17)	0.006	1.12 (1.04–1.20)	0.002
Grade								
I/II	Ref		Ref		Ref		Ref	
III/IV	1.45 (1.30–1.61)	<0.001	1.47 (1.32–1.64)	<0.001	1.48 (1.30–1.68)	<0.001	1.52 (1.34–1.72)	<0.001
Unknown	1.13 (1.04–1.22)	0.002	1.13 (1.04–1.22)	0.002	1.14 (1.04–1.25)	0.006	1.17 (1.07–1.29)	0.001
Tumor site								
Head	Ref		Ref		Ref		Ref	
Body/Tail	0.96 (0.89–1.03)	0.217	0.99 (0.92–1.06)	0.806	0.93 (0.86–1.01)	0.083	0.95 (0.88–1.03)	0.241
Unknown/Over	1.01 (0.94–1.08)	0.872	0.98 (0.92–1.06)	0.656	1.02 (0.94–1.10)	0.707	1.01 (0.93–1.10)	0.847
N stage								
N0	Ref		Ref		Ref		Ref	
N1	1.10 (1.03–1.17)	0.002	1.12 (1.05–1.19)	<0.001	1.10 (1.03–1.18)	0.007	1.11 (1.04–1.19)	0.003
NX	1.14 (1.02–1.29)	0.026	1.07 (0.95–1.20)	0.279	1.08 (0.94–1.26)	0.279	1.03 (0.89–1.19)	0.732
Chemotherapy								
No	Ref		Ref		Ref		Ref	
Yes	0.57 (0.53–0.61)	<0.001	0.64 (0.59–0.68)	<0.001	0.65 (0.58–0.74)	<0.001	0.69 (0.61–0.79)	<0.001

Using Cox proportional hazards models, hazard ratios (HRs) and 95% CIs were calculated for survival status concerning radiation status. Both nonadjusted and multivariate-adjusted models were implemented. *P*-values <0.05 were considered statistically significant.

RT, radiotherapy; Ref, referent

### Multivariate OS analyses

The results of the multivariate analysis revealed that RT was associated with lower mortality compared to non-RT (HR, 0.86; 95% CI: 0.81–0.91; *P*<0.001; non-RT served as the reference). Similarly, chemotherapy (HR, 0.64; 95% CI: 0.59–0.68) was linked to better outcomes than nonchemotherapy. Increasing age was significantly correlated with higher mortality (≥65 years: HR, 1.19; 95% CI: 1.12–1.25), as was marital status (‘other’ marital status: HR, 1.10; 95% CI: 1.04–1.16; with currently married individuals as the reference). Stage N+ disease (HR, 1.12; 95% CI: 1.05–1.19) was associated with worse outcomes than stage N0 disease. Participants with grade III/IV (HR, 1.47; 95% CI: 1.32–1.64) or unknown grade (HR, 1.13; 95% CI: 1.04–1.22) tumors faced a higher risk of death compared to those with grade I/II in this study.

#### Subgroup analyses

**Figure 3:**
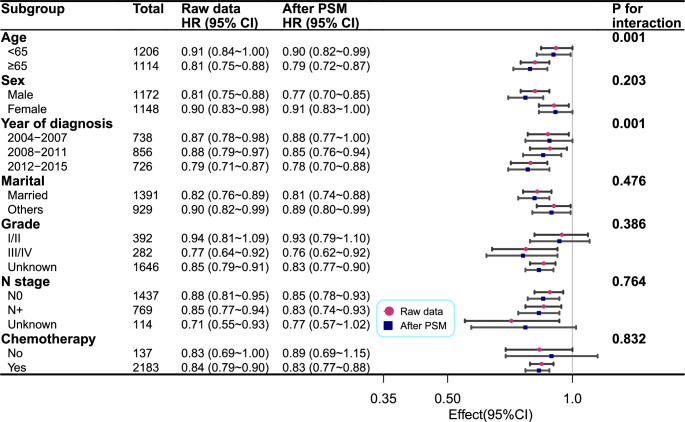
Association between radiotherapy and 3-year mortality according to baseline characteristics before and after PSM. Each stratification was adjusted for potentially confounding factors (i.e. age, sex, year of diagnosis, marital status, race, grade, N stage and chemotherapy), except for the stratification factor itself. HR, hazard ratio; PSM, propensity score matching.

Although we observed statistically significant interactions between chemotherapy and grade in subgroup analyses, the overall HR accurately reflects the effects of RT. Subgroup-specific HRs in this study demonstrated that the practical values indicating the association between RT and OS were consistent across subgroups (Fig. 3). We conducted stratified and interaction analyses to assess the stability of the association between RT and OS within the chemotherapy subgroup. The results remained consistent when analyzing all three models (Table 3). Additionally, we performed a subgroup analysis based on the year of diagnosis in three groups and conducted a Kaplan–Meier survival analysis among patients who received RT (Fig. 2C). The 1-year survival rates were 57.8% (2012–2015), 45.7% (2008–2011), and 41.4% (2004–2007), while the 2-year survival rates were 18.9, 14.0, and 11.8% (*P*<0.0001). Median OS was 14.6, 11.0, and 10.2 months, respectively.

**Table 3:** Multivariable Cox regression analyses of radiotherapy and overall survival by chemotherapy subgroup.

	Unadjusted		Model 1		Model 2		Model 3	
Subgroup	HR (95% CI)	*P*	HR (95% CI)	*P*	HR (95% CI)	*P*	HR (95% CI)	*P*
Nonchemotherapy								
Non-RT (*n*=1100)	1 (Ref)		1 (Ref)		1 (Ref)		1 (Ref)	
RT (*n*=137)	0.82 (0.68–0.98)	0.032	0.83 (0.69–1.00)	0.045	0.84 (0.70–1.00)	0.051	0.83 (0.69–1.00)	0.046
Chemotherapy								
Non-RT (*n*=1993)	1 (Ref)		1 (Ref)		1 (Ref)		1 (Ref)	
RT (*n*=2183)	0.87 (0.81–0.92)	<0.001	0.87 (0.82–0.93)	<0.001	0.84 (0.79–0.89)	<0.001	0.85 (0.80–0.90)	<0.001
P for interaction			0.624		0.785		0.793	

Model 1 adjusted for age and sex.

Model 2 adjusted for age, sex, year of diagnosis, grade, and N stage.

Model 3 adjusted for age, sex, year of diagnosis, grade, N stage, race, marital, and tumor site

#### Sensitivity analyses

PSM analysis was conducted to account for potentially confounding variables, encompassing baseline medical and demographic characteristics. Table 1 presents the descriptive statistics for these variables before and after PSM, illustrating the mitigating effect of this method on potential selection bias. Following the adjustment for pertinent confounders, the administration of RT was significantly associated with improved survival across the entire patient cohort (*P*<0.0001) (Fig. 2B). Furthermore, a statistically significant enhancement in OS was evident for patients treated with RT combined with chemotherapy (CRT) compared to those who received no treatment (Fig. 2D). Multivariate analysis corroborated these findings, revealing a reduced HR in patients receiving RT versus non-RT treatment (HR, 0.84; 95% CI: 0.79–0.90; *P*<0.001) (Table 2). Moreover, we adjusted for propensity score using different statistical methods, and the HR remained consistent (HR= 0.86; 95% CI: 0.81–0.91, *P*<0.001) (Table 4).

**Table 4:** Associations between radiotherapy and overall survival in the crude analysis, multivariable analysis, and propensity-score analyses.

Analysis	HR (95% CI)	*P*
Crude analysis	0.77 (0.72–0.81)	<0.001
Multivariable analysis^a^	0.86 (0.81–0.91)	<0.001
Adjusted for propensity score^b^	0.85 (0.80–0.90)	<0.001
With matched^c^	0.85 (0.79–0.90)	<0.001
With inverse probability weighting^d^	0.86 (0.81–0.91)	<0.001
With SMRW^e^	0.86 (0.81–0.91)	<0.001
With PA^f^	0.85 (0.79–0.91)	<0.001
With Ow^g^	0.85 (0.78–0.93)	<0.001

^a^ Shown is the hazard ratio from the multivariable Cox proportional hazards model, adjusted for all covariates in Table 2.

^b^ Shown is the hazard ratio from a multivariable Cox proportional-hazards model with the same strata and covariates, with additional adjustment for the propensity score.

^c^ Shown is the hazard ratio from a multivariable Cox proportional-hazards model with the same strata and covariates matching according to the propensity score. The analysis included 3846 patients (1918 who received RT and 1918 who did not).

^d^ Shown is the primary analysis with a hazard ratio from the multivariable Cox proportional-hazards model with the same strata and covariates with inverse probability weighting according to the propensity score.

^e^ Shown is the primary analysis with a hazard ratio from the multivariable Cox proportional-hazards model with the same strata and covariates with the standardized mortality ratio weighting according to the propensity score.

^f^ Shown is the primary analysis with a hazard ratio from the multivariable Cox proportional-hazards model with the same strata and covariates with pairwise algorithmic according to the propensity score.

^g^ Shown is the primary analysis with a hazard ratio from the multivariable Cox proportional-hazards model with the same strata and covariates with overlap weight according to the propensity score.

## Discussion

After accounting for patient-related and tumor-related factors, our findings demonstrate that RT is associated with increased median survival compared to non-RT treatment and a reduced mortality risk within 36 months in patients with nonsurgical LAPC. These outcomes were derived from traditional multivariate model analysis. Despite indications of subgroup interactions concerning chemotherapy and grade, sensitivity analysis confirms the enduring benefit of radiation. This study endorses the use of radiation as a means to enhance survival in a substantial, representative observational cohort from a real-world context^16–19^. However, effective RT and definitive guidelines for individuals with non-surgical LAPC remain elusive (Table 1).

Current research focuses mainly on conversion therapy^20^. Combinations of treatments such as RT, chemotherapy and immunotherapy are proving to be safe^21,22^. New biomarkers and RT techniques are also being investigated^23,24^. The integrative treatment model of induction therapy + conversion surgery + adjuvant therapy is becoming one of the standard treatment modalities for LAPC. The importance of the integrated treatment modality is receiving increasing attention. Adjuvant chemotherapy in patients who have undergone successful surgery after conversion therapy is also of interest^25^. Our study also found that 1 in 10 patients (967/9417) underwent surgery, although not exactly radical surgery, which is still encouraging news because the goal of treatment for LAPC is to achieve conversion surgery. But it should also be recognized that there is still a majority of patients who cannot achieve conversion surgery with neoadjuvant therapy. This is the brutal truth. However, what should be the clinical treatment for this group of patients? It is worthy of further consideration. We believe that this study may have brought a lesson in treatment modalities for this group of patients.

Chemotherapy currently stands as the primary therapy for non-surgical LAPC^6,26–29^; nevertheless, even after combined chemotherapy, survival outcomes remain unfavorable^30–33^. Our findings align with prior research, indicating that radiation can extend survival in individuals who are ineligible for resection, though cure rates remain modest. The FFCD/SFCO study^34^ established single-agent gemcitabine chemotherapy as the first-line treatment for LAPC. However, the ECOG trial^35^ and the LAP07 trial^29^ yielded conflicting results regarding whether gemcitabine-based chemotherapy combined with RT offers additional benefits to patients with LAPC. The ECOG trial was a prospective randomized study that assessed the best single agent for the disease (gemcitabine) in conjunction with concurrent RT for LAPC, demonstrating an improvement in median OS (11.1 months vs. 9.2 months) without a significant increase in grade 3–4 side effects. Our research confirms this result (Fig. 2D). In contrast, the LAP07 trial indicated that the chemoradiotherapy group did not enhance OS compared to the gemcitabine-alone group (11.9 months vs. 13.6 months), but it did show significantly higher progression-free survival, suggesting that chemoradiotherapy led to improved local control rates. The main limitations of the LAP07 trial were that the study population was enrolled between 2008 and 2011, and RT was administered using three-dimensional conformal RT with conventional segmentation patterns (1.8Gy/F) and a low RT equivalent biological dose (54Gy/30F). Since 2011, with the widespread adoption of stereotactic body radiotherapy (SBRT) and novel chemotherapeutic agents in LAPC, OS has markedly improved. SBRT has been gradually introduced into the clinical treatment of patients with non-surgical LAPC, achieving a high rate of local tumor control and clinical benefit. The advantage of SBRT over conventional RT is the precise positioning of the target area and the higher dose per segmentation with a reduced number of segmentations, which reduces damage to surrounding normal tissues while achieving a higher biologically effective dose^36–38^.

This trend was confirmed in our study (Fig. 2C). Patients with LAPC treated with RT achieved a longer median OS when using 2011 as the cutoff point. This suggests that new RT techniques and fractionation patterns, when combined with innovative chemotherapeutic agents, may not only enhance local control rates of LAPC but also improve OS.

The current study boasts notable strengths, characterized by its high generalizability and status as the most comprehensive and exhaustive investigation on this subject to date. However, it is essential to acknowledge the study’s limitations. First and foremost, given the retrospective nature of our study and the constraints imposed by the SEER database, we recognize the potential presence of confounding variables that remain unaccounted for in our analysis. Consequently, the reported association between RT and OS may be influenced by concealed effects stemming from unobserved or unmeasured confounding factors. These encompass patient-specific details concerning physical performance status, hematological test results, liver function, and imaging findings, which are not captured within SEER data but can significantly impact physicians’ treatment decisions and patients’ choices to pursue therapy. Moreover, the poor functional status may correlate with unfavorable outcomes.

Various other factors, particularly treatment-related data, have the potential to yield more precise and accurate HR associated with RT or modify the reported connection between RT and reduced survival. While the multivariate analysis can accommodate potential influences of chemotherapy, alongside patients’ demographic and medical profiles, the incorporation of every hypothetical variable in multivariate studies becomes increasingly challenging. From our standpoint, such an exhaustive analysis, adopting a ‘kitchen-sink’ approach, strays from the core objective of this study. Finally, essential details concerning radiation, encompassing dose, frequency, technique, target volume range, and whether patients both initiated and completed radiation, were not documented within the SEER database. Given the observational database’s constraints, we employed PSM and subgroup analyses to mitigate differences between treatment groups as comprehensively as possible.

## Conclusions

This study aimed to perform a stability analysis within a large, nationally representative observational epidemiological cohort to investigate the effect and efficacy of RT on non-surgical LAPC. Our findings imply that palliative RT should be explored for individuals with nonsurgical LAPC within 3 years of treatment commencement. Prospective randomized controlled clinical trials on this subject remain insufficient, which are essential to corroborate the reported association between RT and OS in patients with nonsurgical LAPC. Chemotherapy and tumor grade were associated with outcomes and treatment modalities in our study and, hence, are of considerable interest for future studies. The current study’s findings will inform prospective, highly informative, and impactful clinical studies and, if verified, medical guidelines and clinical decision-making.

## Ethical approval

As all the data used in this study were derived from publicly available sources within the SEER database, there was no requirement for informed consent or ethical approval.

## Consent

As all the data used in this study were derived from publicly available sources within the SEER database, there was no requirement for informed consent or ethical approval.

## Source of funding

There was no external support for this work.

## Author contribution

Y.D.: conducted data analysis and wrote the manuscript; J.S.: conducted data analysis and modified the manuscript; Z.S.: performed data collection; Y.D.: worked on the data collection; Y.W.: showed data collection and data interpretation; J.S.: drew the figure; L.M.: designed the study and reviewed the manuscript.

## Conflicts of interest disclosure

The authors declare that the research was conducted in the absence of any commercial or financial relationships that could be construed as a potential conflict of interest.

## Research registration unique identifying number (UIN)

As all the data used in this study were derived from publicly available sources within the SEER database, there was no requirement for informed consent or ethical approval.

## Guarantor

Yupeng Di.

## Data availability statement

The data that support the findings of this study are openly available in SEER database at https://seer.cancer.gov/seerstat. The raw data supporting the conclusions of this article will be made available by the authors, without undue reservation.

## Provenance and peer review

Not commissioned, externally peer-reviewed.
